# An updated meta-analysis showed smoking modify the association of *GSTM1* null genotype on the risk of coronary heart disease

**DOI:** 10.1042/BSR20200490

**Published:** 2021-02-12

**Authors:** Yadong Song, Zhilei Shan, Xiaoli Liu, Xiaomin Chen, Cheng Luo, Liangkai Chen, Yimei Wang, Lin Gong, Liegang Liu, Jiansheng Liang

**Affiliations:** 1 Department of Disinfection and Pest Control, Wuhan Centers for Disease Prevention and Control, Wuhan, Hubei, People's Republic of China; 2 Wuhan Healthcare-Associated Infection Management Quality Control Center, Wuhan, Hubei, People's Republic of China; 3 Department of Nutrition and Food Hygiene, Hubei Key Laboratory of Food Nutrition and Safety, Tongji Medical College, Hua Zhong University of Science and Technology, People's Republic of China; 4 Ministry of Education Key Lab of Environment and Health, School of Public Health, Tongji Medical College, People's Republic of China

**Keywords:** Coronary heart disease, Genetic polymorphism, GSTM1, Meta-analysis, Smoking

## Abstract

**Background** Oxidative stress is considered to be involved in the pathogenesis of coronary heart disease (CHD). Glutathione-S-transferase (GST) enzymes play important roles in antioxidant defenses and may influence CHD risk. The present meta-analysis was performed to investigate the link between glutathione S-transferase M1 (*GSTM1*) null genotype and CHD and to get a precise evaluation of interaction between *GSTM1* null genotype and smoking by the case-only design.

**Methods** PubMed and EMBASE databases were searched through 15 December 2020 to retrieve articles. Odds ratios (ORs) were pooled using either fixed-effects or random-effects models.

**Results** Thirty-seven studies showed that *GSTM1* null genotype was associated with risk of CHD in total population, Caucasians and Asians (for total population, OR = 1.38, 95% confidence interval (CI): 1.15, 1.65; for Caucasians, OR = 1.34, 95% CI: 1.04, 1.72; for Asians, OR = 1.40, 95% CI: 1.11, 1.77). After adjustment for heterogeneity, these relationships were still significant. After adjustment for heterogeneity, case-only analysis of 11 studies showed a positive multiplicative interaction between *GSTM1* null genotype and smoking (ever smoking vs. never smoking) (OR = 1.27, 95% CI: 1.08, 1.50; *I^2^* = 0%, *P*=0.553).

**Conclusions** The overall results indicated that *GSTM1* null genotype was associated with a higher risk of CHD, and the association may be affected by smoking status. This is the first meta-analysis to prove a positive effect of the interaction between *GSTM1* null genotype and smoking status on the risk of CHD. Well-designed studies are needed to investigate the possible gene–gene or gene–environment interactions.

## Introduction

Coronary heart disease (CHD) is the leading cause of mortality and a major cause of morbidity and disability all over the world [1,2]. CHD is an extremely multifactorial disease, which is influenced by both complex genetic and multiple environmental factors, as well as their interactions.

There is compelling evidence that cigarette smoking is one of the strong risk factors for CHD. Multiple chemicals in cigarette smoke can cause endothelial dysfunction, smooth muscle cell proliferation, generation of reactive oxygen species (ROS) and DNA damage, which can lead to atherosclerosis and, hence, CHD [3–6]. However, only a small number of smokers ultimately develop CHD. The differential susceptibility to CHD among smokers may be influenced by polymorphisms in genes encoding the metabolic enzymes, which play important roles in the detoxification of toxic chemicals generated by smoking.

The glutathione S-transferases (GSTs) are an important family of phase II isoenzymes which can detoxify electrophilic compounds generated by smoking, including toxins, DNA adducts, and carcinogens, mainly by changing them to harmless products through conjugation to glutathione [7,8]. In addition, GSTs can modulate the induction of other proteins and enzymes which are important for cellular functions, such as DNA repair [9].

Human cytosolic GST enzymes which comprise multiple isoenzymes are divided into eight separate classes: GSTM (mu), GSTP (pi), GSTT (theta), GSTA (alpha), GSTK (kappa), GSTO (omega), GSTS (sigma), and GSTZ (zeta) [10]. The Mu class of GSTs is encoded by the glutathione S-transferase M1 (*GSTM1*) gene, which is mapped to chromosome 1p13.3. Three alleles of the *GSTM1* locus have been identified: *GSTM1* null and two others (*GSTM1a* and *GSTM1b*) that differ by C→G substitution at base position 534. The C→G substitution leads to the substitution Lys→Asn at amino acid 172 [11]. Persons with homozygous deletions of the *GSTM1* locus have been associated with no enzymatic functional activity and increased vulnerability to cytogenetic damage [12,13], and thus it was hypothesized to be linked with risk of CHD [14].

Our previous meta-analysis have proved that the null genotype of GSTT1 was associated with an increased risk of CHD [15]. Indeed, a great number of studies have investigated the association between *GSTM1* genetic polymorphism and risk of CHD. However, results have been inconsistent [16–50], and the interaction between *GSTM1* null genotype and smoking is unclear. To our knowledge, two previous meta-analyses [51,52] investigating the association between *GSTM1* null genotype and CHD risk have yielded contradictory findings. One previous meta-analysis [51] reported that *GSTM1* null genotype may be an independent risk factor for CHD and the other meta-analysis [52] indicated that a negative association exists between *GSTM1* null genotype and CHD risk. To help clarify the inconsistent findings, we conducted a meta-analysis to investigate the association between polymorphism of *GSTM1* and CHD risk. Furthermore, we performed a case-only design to get a more precise evaluation of interaction between *GSTM1* null genotype and smoking on CHD risk.

## Materials and methods

### Search strategy and selection criteria

We searched electronic databases, including PubMed and Embase, for all articles published through 15 December 2020, which had investigated the association between *GSTM1* genotype (null genotype vs. wildtype) and the risk of CHD. The terms used for searching included glutathione S-transferase, GST, *GSTM1*; gene, polymorphism; and coronary heart disease, CHD, myocardial infarction, MI, coronary artery disease, CAD, ischemic heart disease. References cited in retrieved articles and published review articles were also screened to identify additional publications. If there were several publications from the same study, we selected the most complete or most recent publication for meta-analyses. To minimize potential publication bias, studies without any special restriction were included.

The inclusion criteria were: (i) studies with case–control design examining the association between CHD risk and polymorphism of *GSTM1*; (ii) presenting original data for the calculation of odds ratios (ORs) with corresponding 95% confidence intervals (95% CIs); (iii) clear definition of CHD. The exclusion criteria were: (i) case-only studies, animal studies, simply commentaries, case reports and review articles; (ii) studies with other genotypes of GST or other disease.

### Data extraction and quality assessment

Characteristics abstracted from the articles included the name of the first author, year of publication, country, ethnicity, genotyping method, control source, number of cases, number of controls, cases null, controls null, Hardy–Weinberg equilibrium (i.e., the genotype distribution in the control population were in accordance with Hardy–Weinberg equilibrium: yes, no, not available), and adjustment covariates. When specific results were not reported, we used available tabular data to calculate them. When data were unavailable, we contacted the corresponding author by email for additional information. Different ethnicities were categorized as Caucasian, Asian, and Mixed. The bibliographic search, data extraction, and quality assessment were conducted independently by two authors, and any disagreements were resolved by consensus with a third investigator.

We assessed quality of included studies based on Newcastle–Ottawa Scale (NOS) [53]. The NOS is an 8-item instrument, and the detail of NOS grading standard is listed as follows: (i) selection, included adequate definition of patient cases, representativeness of patients cases, selection of controls, definition of controls, total score: 4; (ii) comparability, included Control for important factor or additional factor, total score: 2; (iii) exposure (case–control studies), included ascertainment of exposure (blinding), same method of ascertainment for participants, non-response rate, total score: 3. A star system of the NOS (range, 0–9 stars) has been developed for quality assessment (Supplementary Table S1). The mean value for all included studies was 7 stars.

### Statistical analyses

Based on the genotype frequencies, crude ORs corresponding to 95% CI were calculated to measure the association between *GSTM1* null genotype and risk of CHD. Cochran's χ^2^ based Q-statistic test and *I^2^* test were performed to precisely assess possible heterogeneity, which quantified between-study heterogeneity irrespective of the number of studies [54]. If heterogeneity was considered significant at *P*<0.1 (Cochran's χ^2^ based Q-statistic test), a random-effects model (DerSimonian–Laird method) [55] was used to calculate the pooled ORs. Otherwise, the fixed-effect model [56] was conducted [57,58]. An *I^2^* value less than 50% was considered to indicate low heterogeneity [59]. The meta-regression was performed to study the source of between-study heterogeneity [60]. The introduction of covariates for assessment of heterogeneity sources were publication year, ethnicity, sample size, and control source. If there was heterogeneity between studies, sources of heterogeneity were also investigated by stratified meta-analyses based on ethnicity (Asian, Caucasian); source of controls (population-based, hospital-based); sample size (number of cases <600 or >600). Sensitivity analysis, removing one study at a time, was also performed to evaluate the stability of the results. Besides, Galbraith plot was also conducted to spot the outlier as the possibly major source of between-study heterogeneity [61]. The outliers were considered as the possible major source of heterogeneity, and further meta-analysis after adjustment for heterogeneity was performed by excluding these studies. The potential publication bias was investigated by means of Begg's funnel plot and Egger's test [62].

To investigate the multiplicative interaction between *GSTM1* null genotype and smoking (ever smoking vs. never smoking) on CHD risk, we also performed a case-only design in present meta-analyses [63,64]. All analyses were performed using Stata, version 11.0 (StataCorp, College Station, Texas). All tests were two-sided with a significance level of 0.05.

## Results

### Characteristics of the included studies

**Figure 1:**
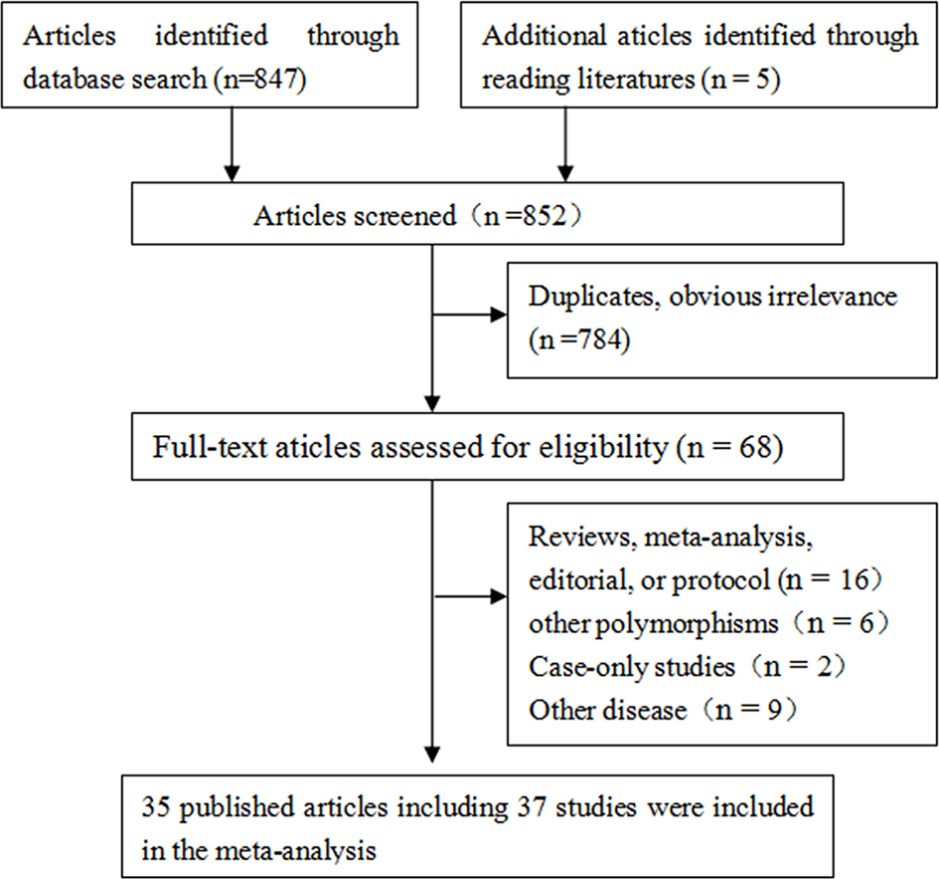
Flow chart depicting exclusion/inclusion of individual articles for meta-analysis

In total, 37 studies from 35 articles matching the search terms, comprising 16684 cases and 36510 controls, were retrieved from databases. A flow chart describing the exclusion/inclusion of individual articles has been presented as Figure 1. A total of 852 articles were found with our search criteria. One article contained three individual case–control studies [38] and one article was published in Chinese [36]. Table 1 showed characteristics of these 37 studies, 23 [16–23,26–29,33–35,37,38,41,44,45,48] were from Caucasian population, 13 [24,25,30,31,36,39,40,42,43,46,47,49,50] were from Asians, and 1 [32] was Mixed ethnicity. The number of cases varied from 29 to 2360, with a mean of 451, and the number of controls varied from 30 to 9099, with a mean of 988 (Table 1).

**Table 1:** Characteristics of studies included in a meta-analysis of *GSTM1* null genotype and CHD risk

First author	Year	Country	Ethnicity	Genotyping method	Control source	Number of cases	Number of controls	Cases null	Controls null	Hardy– Weinberg Equilibrium	Adjustment covariates
Evans [16]	1996	Saudi Arabia	Caucasian	PCR	PB	90	884	57	504	NA	NA
Wilson [17]	2000	U.K.	Caucasian	PCR	PB	356	187	191	107	NA	NA
Li [18]	2000	U.S.A.	Caucasian	PCR	PB	400	890	178	354	NA	Age, sex, race, LDL, HDL, hypertension and diabetes
Wang [20]	2001	U.S.A.	Caucasian	PCR	HB	612	256	343	153	NA	NA
Salama [19]	2002	U.S.A.	Caucasian	PCR	PB	120	90	45	33	NA	NA
Wilson [24]	2003	U.K.	Asian	PCR	PB	170	203	70	107	NA	NA
Palmer [23]	2003	U.K.	Caucasian	PCR	HB	51	57	40	35	NA	Age, smoking, duration of disease, sex, HDL, glucose, triglycerides, and blood pressure
Olshan [22]	2003	U.S.A.	Caucasian	PCR	PB	526	868	252	352	NA	Age, sex and race
Masetti [21]	2003	Italy	Caucasian	PCR	HB	308	122	163	66	NA	NA
Girisha [25]	2004	India	Asian	PCR	PB	197	198	46	41	Yes	NA
Tamer [26]	2004	Turkey	Caucasian	RT-PCR	PB	148	247	67	103	NA	NA
Hayek [28]	2006	U.K.	Caucasian	PCR	PB	193	2399	88	1142	NA	NA
Abu-Amero [27]	2006	Saudi Arabia	Caucasian	PCR	HB	1054	762	655	117	NA	Hypertension, cholesterol, obesity, smoking
Cornelis [29]	2007	Canada	Caucasian	PCR	PB	2042	2042	980	531	NA	Age, sex, area, smoking, waist-to-hip ratio, income, physical activity, history of diabetes and hypertension, intake of alcohol, and energy adjusted saturated fat and folate
Kim [30]	2008	Korea	Asian	PCR	HB	356	336	198	191	NA	Age, sex, hypertension, DM, BMI and lipid profile
Wang [31]	2008	China	Asian	PCR	HB	277	277	89	59	Yes	Diabetes, hypertension, smoking status
Martin [34]	2009	U.S.A.	Caucasian	PCR	PB	67	63	41	19	NA	NA
Manfredi [33]	2009	Italy	Caucasian	PCR	HB	184	47	108	18	NA	NA
Maciel [32]	2009	Brazil	Mixed	PCR	PB	869	1573	557	789	NA	NA
Ramprasath [39]	2011	India	Asian	PCR	HB	290	492	128	150	NA	NA
Bazo [35]	2011	Brazil	Caucasian	PCR	HB	297	100	160	44	NA	NA
Singh [40]	2011	India	Asian	PCR	PB	230	300	56	65	NA	Age, sex, BMI, smoking, alcohol, food habit, lipid profile and fasting glucose
Nomani [37]	2011	Iran	Caucasian	PCR	HB	209	108	100	57	NA	NA
Norskov CCHS [38]	2011	Denmark	Caucasian	RT-PCR	PB	1769	8425	921	4414	Yes	NA
Norskov CGPS [38]	2011	Denmark	Caucasian	RT-PCR	PB	801	9099	411	4738	Yes	NA
Norskov CIDHS [38]	2011	Denmark	Caucasian	RT-PCR	PB	2360	4160	1203	2210	NA	NA
Zhang [36]	2011	China	Asian	PCR	PB	255	145	120	46	NA	NA
Taspinar [44]	2012	Turkey	Caucasian	PCR	PB	122	142	51	66	NA	Age, gender, family history, smoking status, and diabetes
Kariz [41]	2012	Slovenia	Caucasian	PCR	HB	206	257	64	91	NA	Age, gender, diabetes, BMI, smoking, lipid parameters
Lakshmi [42]	2012	India	Asian	PCR	PB	350	282	68	54	Yes	Age, BMI, gender, diabetes, family history of CAD
Phulukdaree [43]	2012	South Africa	Asian	PCR	PB	102	100	37	18	Yes	NA
Cora [45]	2013	Turkey	Caucasian	PCR	PB	324	296	182	143	NA	Age, sex, smoking, diabetes, hypertension, family history, lipid profile
Yeh [46]	2013	Taiwan	Asian	PCR	HB	458	209	253	121	Yes	Age, sex, cigarette smoking, alcohol use, diabetes mellitus, and levels of serum total cholesterol and high-density lipoprotein cholesterol
Kadıoğlu [48]	2016	Turkey	Caucasian	PCR-RFLP	PB	29	30	17	14	Yes	Age, gender, hypertension and smoking habit
Bhat [47]	2016	India	Asian	PCR	PB	200	200	62	36	NA	Age, gender, body mass index, alcohol, total cholesterol, hypertension and family history of CAD
Mir [49]	2017	India	Asian	PCR	PB	100	100	42	26	Yes	NA
Bhatti [50]	2018	India	Asian	PCR	PB	562	564	217	127	NA	NA

Abbreviations: AMI, acute myocardial infarction; AR, atherosclerosis; BMI, body mass index; CAD, coronary artery disease; HB, hospital-based; IHD, ischemic heart disease; MI, myocardial infarction; NA, not available; PB, population-based; PCR, polymerase chain reaction; RT-PCR, reverse transcription PCR.

### GSTM1

**Figure 2:**
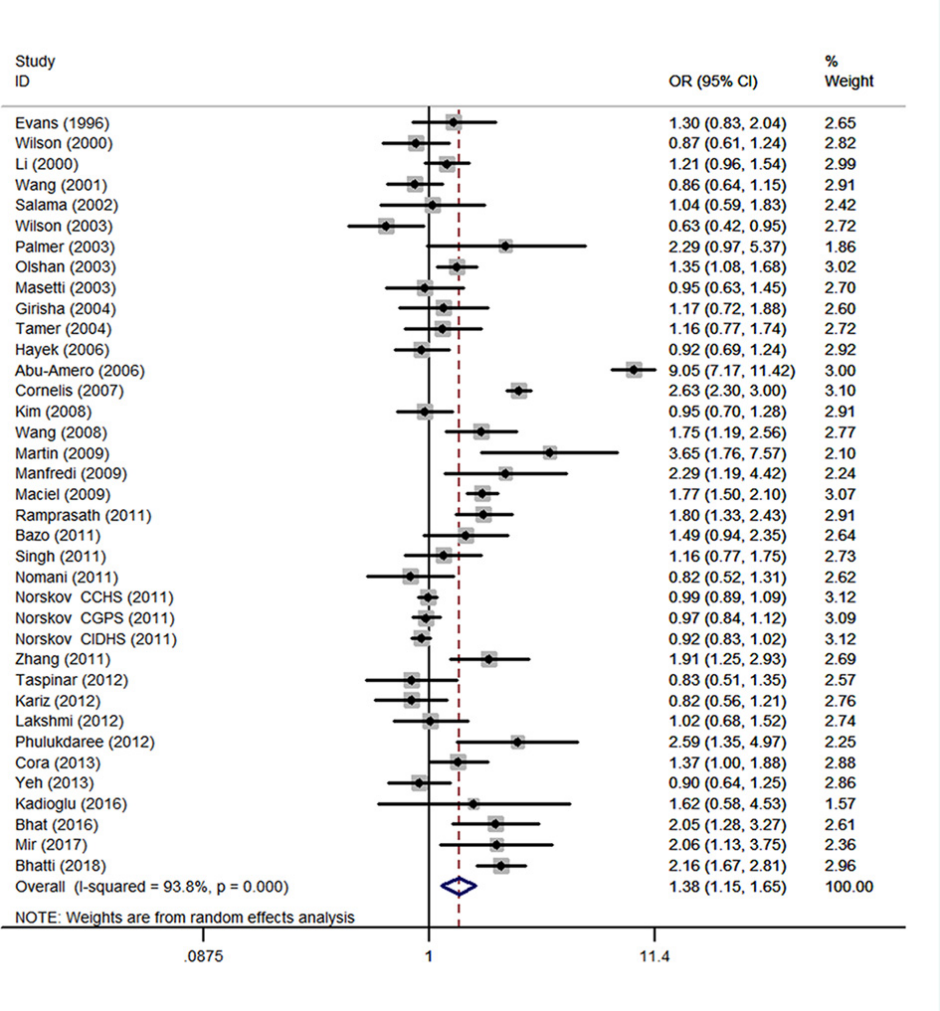
Meta-analysis of *GSTM1* null genotype associated with CHD Each box represents the OR point estimate, and its area is proportional to the weight of the study. The diamond represents the overall summary estimate, with CI represented by its width.

**Figure 3:**
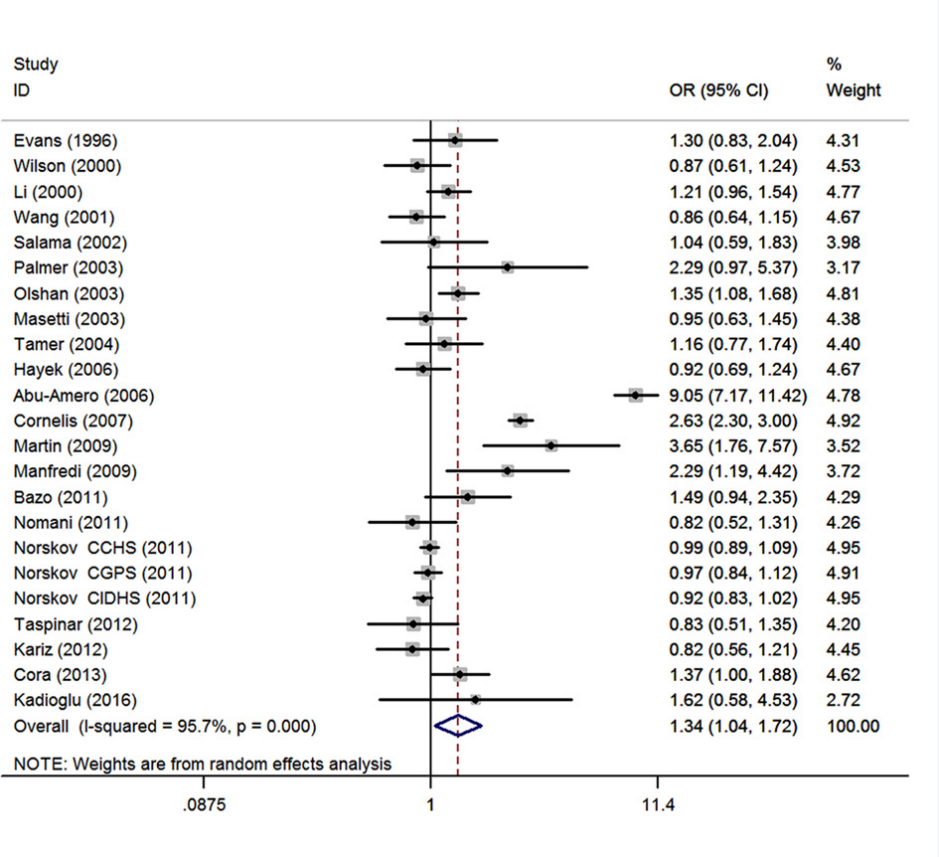
Meta-analysis of Caucasian studies Each box represents the OR point estimate, and its area is proportional to the weight of the study. The diamond represents the overall summary estimate, with CI represented by its width.

A total of 37 studies with 16684 cases and 36510 controls were retrieved based on the search criteria for CHD susceptibility related to the *GSTM1* null polymorphism. Heterogeneity between studies was suggested (*I^2^* = 93.8%; *P*<0.001), thus the random-effects model was used to pool data. The results indicated that the *GSTM1* null genotype was significantly associated with CHD (OR = 1.38, 95% CI: 1.15, 1.65) (Figure 2). There was no evidence of publication bias (Begg's test, *P*=0.097; Egger's test, *P*=0.499 (Table 2). The meta-regression was conducted with the introduction of covariates including publication year, ethnicity, sample size, and control source. However, no covariate was identified as a potential source of between-study heterogeneity for any comparison. Sensitivity analyses indicated that the study by Abu-Amero et al. [27] was the main origin of heterogeneity in overall OR. After exclusion of the study [27], the heterogeneity was decreased (*I^2^* = 88.6%). Besides, sensitivity analyses which yielded a range of ORs from 1.28 (95% CI: 1.12, 1.48) to 1.40 (1.17, 1.69) suggested that the results of this meta-analysis are stable. For meta-analysis of total studies, fifteen studies were spotted by Galbraith plot as possible major sources of heterogeneity [20,24,27,29,32–34,36,38,39,41,43,50]. There was no obvious between-study heterogeneity among remaining studies (*I^2^* = 40.2%; *P*=0.027), and meta-analysis showed *GSTM1* null genotype was also associated with increased risk of CHD (OR = 1.17, 95% CI: 1.05, 1.31) (Table 2). By stratifying the analysis by ethnicity, an OR of 1.34 (95% CI: 1.04, 1.72; *I^2^* = 95.9%, *P*<0.001) (Figure 3) and 1.40 (95% CI: 1.11, 1.77; *I^2^* = 78.6%, *P*<0.001) (Table 2) resulted in null genotype, among Caucasians and Asians, respectively. For meta-analysis of Caucasian studies, ten studies were spotted by Galbraith plot as possible major sources of heterogeneity [20,27–29,33,34,38,41]. After adjustment for heterogeneity by excluding these studies, the association was still significant in Caucasians (OR = 1.18, 95% CI: 1.07, 1.31; *I^2^* = 18.1%, *P*=0.261). For meta-analysis of Asian studies, four studies were spotted by Galbraith plot as possible major sources of heterogeneity [24,30,46,50]. After adjustment for heterogeneity by excluding these studies, the association was still significant in Asians (OR = 1.60, 95% CI: 1.32, 1.95; *I^2^* = 44.2%, *P*=0.073). Subgroup analysis by source of controls yield an OR of 1.47 (95% CI: 0.86, 2.51; *I^2^* = 96.1%, *P*<0.001) and 1.33 (95% CI: 1.11, 1.58; *I^2^* = 91.2%, *P*<0.001) resulted for null genotype, among hospital-based controls and healthy controls, respectively (Table 2). Stratified by sample size showed that the combined ORs were 1.32 (95% CI: 1.09, 1.61) for studies with the sample size < 600 and 1.40 (1.07, 1.84) for studies with the sample size > 600 (Table 2). Among smokers in 14 studies, people with the *GSTM1* null genotype had an increased CHD risk with an OR of 1.64 (95% CI: 1.12, 2.40; *I^2^* = 82.2%, *P*<0.001) (Table 2). Among non-smokers in 11 studies, people with the *GSTM1* null genotype was not associated with CHD risk (OR = 1.26, 95% CI: 0.70, 2.27; *I^2^* = 94.2%, *P*<0.001) (Table 2).

**Table 2:** Subgroup analyses of studies included in a meta-analysis of *GSTM1* null genotype and CHD risk

Null versus present	Studies	Cases/controls	OR (95% CI)	Heterogeneity		Model	*P* for Begg's test	*P* for Egger's test
				*I^2^*	*P_H_*			
Total studies	37	16684/36510	1.38 (1.15, 1.69)	93.8%	<0.001	Random	0.097	0.499
Total studies (adjustment for heterogeneity^1^)	22	5341/8322	1.17 (1.05, 1.31)	40.2%	0.027	Random	0.236	0.424
Smoker	14	2249/1300	1.64 (1.12, 2.40)	82.2%	<0.001	Random	0.189	0.387
Non-smoker	11	1962/2195	1.26 (0.70, 2.27)	94.2%	<0.001	Random	0.755	0.043
Ethnicity								
Caucasians	23	12268/31531	1.34 (1.04, 1.72)	95.7%	<0.001	Random	0.045	0.605
Caucasians (adjustment for heterogeneity^2^)	13	2980/4021	1.18 (1.07, 1.31)	18.1%	0.261	Fixed	1.00	0.763
Asians	13	3547/3406	1.40 (1.11, 1.77)	78.6%	<0.001	Random	0.583	0.903
Asians (adjustment for heterogeneity^3^)	9	2001/2094	1.60 (1.32, 1.95)	44.2%	0.073	Random	0.348	0.557
Source of controls								
HB	12	4302/3023	1.47 (0.86, 2.51)	96.10%	<0.001	Random	0.244	0.238
PB	25	12382/33487	1.33 (1.11, 1.58)	91.20%	<0.001	Random	0.199	0.418
Sample size								
<600	20	3628/2973	1.32 (1.09, 1.61)	68.5%	<0.001	Random	0.041	0.016
>600	17	13056/33537	1.40 (1.07, 1.84)	96.9%	<0.001	Random	0.650	0.472

Abbreviations: HB, hospital based; PB, population based.

*P_H_*: *P*-value based on Q test for between-study heterogeneity.

1 Adjustment for heterogeneity was performed by excluding 15 studies as the outliers and the possible major source of heterogeneity.

2 Adjustment for heterogeneity was performed by excluding 10 studies as the outliers and the possible major source of heterogeneity.

3 Adjustment for heterogeneity was performed by excluding 4 studies as the outliers and the possible major source of heterogeneity.

### Smoking

**Figure 4:**
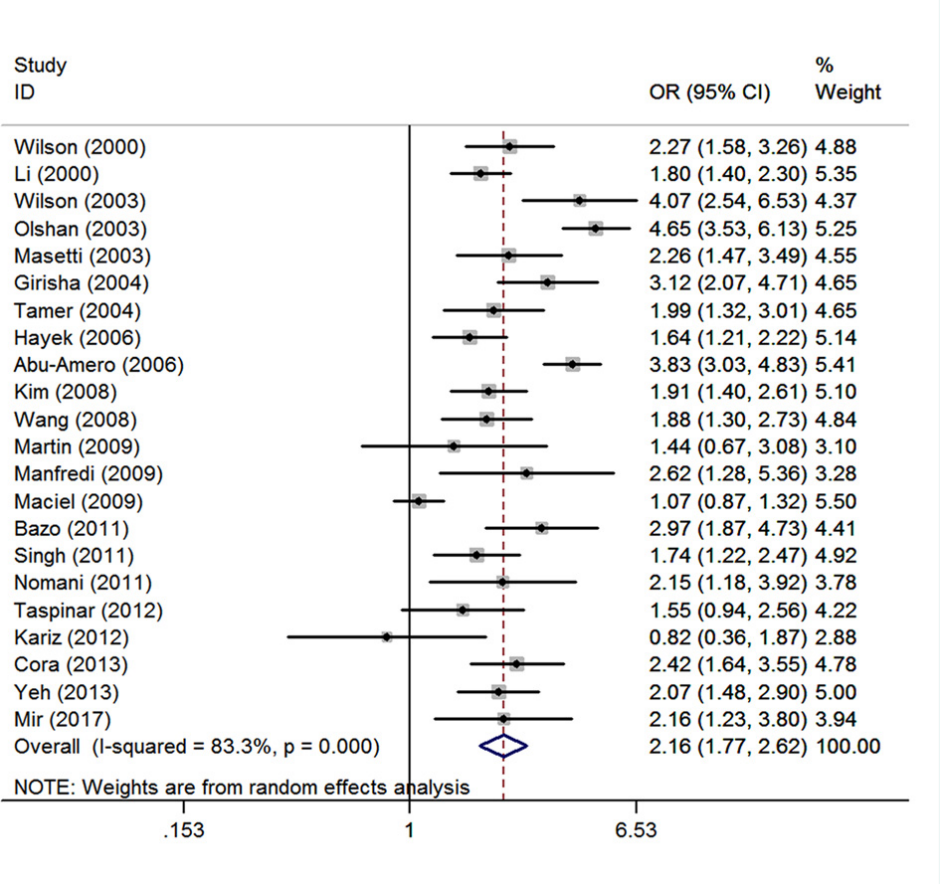
Summary estimate (ORs and 95% CI) of CHD risk associated with smoking Each box represents the OR point estimate, and its area is proportional to the weight of the study. The diamond represents the overall summary estimate, with CI represented by its width.

There are 22 studies [17,18,21,22,24–28,30–35,37,40,41,44–46,49] comprising 6816 CHD cases and 9822 controls. There was obvious between-study heterogeneity was detected among total 22 studies (*I^2^* = 83.3%; *P<*0.001), and thus the random-effects model yielded an OR of 2.16 (1.77, 2.62) (Figure 4). The Begg's test (*P*=0.735) and Egger's test (*P*=0.808) showed no publication bias. After the exclusion of 4 studies [22,24,27,32] spotted by Galbraith plot as possible major sources of heterogeneity, there was no obvious between-study heterogeneity among those remained studies (*I^2^* = 10.9%; *P*=0.324). Thus, the fixed-effects model was used to pool the ORs, and the result was not substantially changed (OR = 2.00, 95% CI: 1.82, 2.20). We performed a sensitivity analysis by omitting one study at a time, which yielded a range of ORs from 1.95 (95% CI: 1.77, 2.15) to 2.04 (1.84, 2.27).

### *GSTM1*-smoking interplay

**Figure 5:**
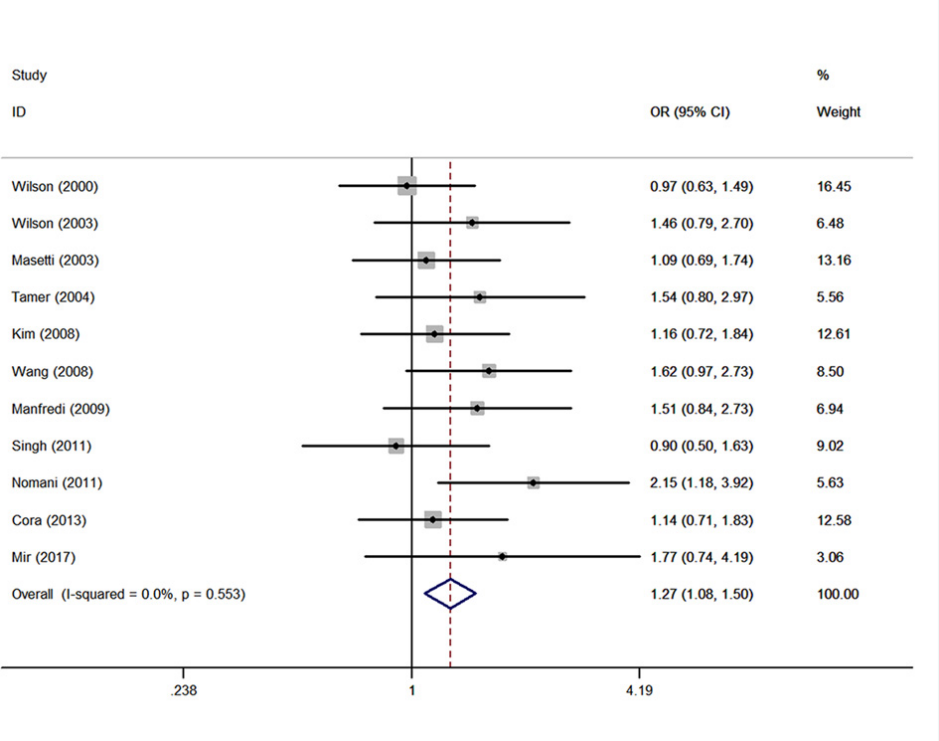
Summary estimate (ORs and 95% CI) of the effect of interaction between the *GSTM1* null genotype and smoking (ever vs. never) on CHD risk after adjustment for heterogeneity Each box represents the OR point estimate, and its area is proportional to the weight of the study. The diamond represents the overall summary estimate, with CI represented by its width.

Twelve studies [17,21,24,26,27,30,31,33,37,40,45,49] included in the case-only analysis revealed a positive effect of the interaction between the *GSTM1* null genotype and smoking status (ever smoking vs. never smoking) (OR = 1.49, 95% CI: 1.06, 2.08; *I^2^*= 80.9%; *P*<0.001). After omitting one study [27] which was spotted by Galbraith plot as the major sources of heterogeneity, the interaction between the *GSTM1* null genotype and smoking on CHD risk was also statistically significant (OR = 1.27, 95% CI: 1.08, 1.50; *I^2^* = 0%, *P*=0.553) (Figure 5).

## Discussion

The current meta-analysis provided a comprehensive evaluation of the association between *GSTM1* genetic polymorphism with risk of CHD. Moreover, to our knowledge, this is the first case-only designed analysis to prove a positive effect of the interaction between *GSTM1* null genotype and smoking on CHD risk.

Two previous meta-analyses were performed to evaluated the association between GSTM1 genetic polymorphism with risk of CHD.The first one, performed in 2010 by Wang et al. [51], included 8020 cases and 11501 controls from 19 studies. They found a significant association between null polymorphism of GSTM1 and CHD risk. Afterwards, an updated meta-analysis conducted by Zhou et al. [52] showed that GSTM1 null genotype was not associated with increased risk of CHD in total population. In the present study, we identified 37 eligible studies, including 16684 CHD cases and 36510 controls, which could provide sufficient statistic power. Compared with previous meta-analyses, more than 11 relevant studies [36,37,39–42,45,47–50] were included in our analysis but not in theirs. Our meta-analysis showed that the GSTM1 null genotype was associated with a statistically elevated risk of CHD (OR = 1.38, 95% CI: 1.15, 1.65), which was consistent with the study by Wang et al. [51], but not the study by Zhou et al. [52]. After adjustment for heterogeneity by excluding these studies spotted by Galbraith plot, the results were still stable. By stratifying the analysis according to ethnicity, two previous meta-analyses [51,52] both found that GSTM1 null genotype was not associated with the risk of CHD for either Caucasians or Asians. However, our meta-analysis showed that the null genotype of GSTM1 may be associated with a higher risk of CHD in both Caucasians and Asians, which was inconsistent with two previous meta-analysis [51,52]. The results were still stable after adjustment for heterogeneity (Table 2). Two previous meta-analyses were relatively small and insufficient data were available for more exhaustive subgroup analysis. Among smokers in 14 studies, individuals with the null genotype of GSTM1 had a significantly increased CHD risk, which was consistent with two previous meta-analyses [51,52].

When interpreting the results of meta-analyses, heterogeneity assessment is necessary [65,66]. The *I^2^* values surpassed the threshold of 50% in the present meta-analyses, indicating the presence of heterogeneity and insufficient power [59]. Meta-analyses might miss true effects when even modest between-study heterogeneity is present. Besides, low quality designed studies may result in incorrect conclusions [66]. In the present study, Galbraith plot was conducted to detect the outliers as the possible studies with low quality design and sensitivity analysis was further performed by omitting studies potted by Galbraith plot's method as the outliers. Fifteen studies were detected by Galbraith plot as possible major sources of heterogeneity in total studies, and ten studies were spotted by Galbraith plot as the possibly major sources of heterogeneity in Caucasian studies. When omitting those studies, the between-study heterogeneity decreased and there was no obvious heterogeneity among the remained studies (Table 2), which proved that those studies result in the heterogeneity. After adjustment for heterogeneity, meta-analyses showed that *GSTM1* null genotype still increased risk of CHD in total population, Caucasians and Asians, respectively (Table 2). Errors and biases which led to heterogeneity were not known. Furthermore, there was limited knowledge on how much heterogeneity represented a true difference in genetic effects among different populations. Further studies need to focus on exploring the sources of heterogeneity.

Considering that CHD is a multifactorial trait and the impact of the GSTs on the progress of CHD may be modulated by age, gender and some other environmental and genetic influences, several subgroup meta-analyses were conducted in the present meta-analysis. In racial subgroups, meta-analysis showed *GSTM1* null genotype increased risk of CHD both in Caucasians and in Asians. When stratifying by control source, significant association between null genotype of *GSTM1* and CHD risk was observed population-based studies but not in hospital-based studies. By considering control source subgroups, Wang et al. [51] reported that GSTM1 null genotype was not associated with the risk of CHD in both population-based controls and hospital controls. The results may be biased by studies conducted by Abu-Amero et al. [27], Cornelis et al. [29], Nomani et al. [37], and Ramprasath et al. [39], because these studies included high-risk people with diabetes mellitus, hypertension, or obesity. Besides, what also needs to be pointed out is that the result should be interpreted with caution because of the relatively small sample size.

In present meta-analyses, the results suggest a positive multiplicative interaction (i.e., OR > 1) between smoking status and the *GSTM1* null genotype on CHD risk. People with the *GSTM1* null genotype were associated with CHD risk among smokers, but not among non-smokers in the present study. Cigarette smoking is a pro-inflammatory stimulus, and it is an important risk factor for CHD. Multiple chemicals in cigarette smoke can induce oxidative stress that results in smooth muscle cell proliferation, inflammation, vascular dysfunction DNA damage, and lipid peroxidation, which lead to atherosclerosis, and hence, CHD [3,4,6]. Animal experiments have proved that aromatic amines and polycyclic aromatic hydrocarbons (PAHs) in tobacco smoke can cause atherosclerotic lesions [67,68]. Moreover, DNA damage is present in cardiovascular disease patients [69]. Components in cigarette smoke can induce DNA adducts mitochondrial DNA damage in vascular cells [70] and DNA adducts in target tissues [5]. Oxidative stress and DNA damage play important roles in pathogenesis of atherosclerosis which is responsible for CHD. GSTs constitute the major defensive antioxidant system against oxidative stress by reducing ROS, which detoxify metabolites produced by oxidative stress and DNA damage within the cell and protect the cells against injury [71,72]. A homozygous deletion (0/0) or null genotype at the *GSTM1* locus is related to enzyme function loss, which may be associated with susceptibility to CHD. Thus, there is biological evidence for the association between CHD risk and *GSTM1* null genotype. The interaction between the *GSTM1* null polymorphism and smoking status suggests that smoking is more detrimental to people who carry the *GSTM1* null genotype. Although we pooled all published studies currently available on this topic, we thought our study was still far from conclusive, because many studies did not stratify the results according to smoking status. Besides, the sample sizes of these studies were small to modest, limiting their statistical power of the individual studies to detect interaction.

This meta-analysis had several limitations. First, the eligibility criteria for inclusion of controls were different. Some studies selected healthy individuals as controls, while the controls in other studies were selected from non-CHD individuals. Thus, selection bias might exist. Second, this meta-analyses were based on unadjusted estimates because many studies did not provide adjusted data. Third, some of these studies had relatively small sample sizes, which decreased their statistical power. Fourth, a possible publication bias may exist because only published studies were included, though there was no evidence of publication bias by visual examination of Begg's funnel plot or test results from Egger's test.

In conclusion, the present study showed that *GSTM1* null genotype seems to be a risk factor for CHD. And the association may be affected by smoking status. The interaction between the *GSTM1* null genotype and smoking status on CHD risk suggests that smoking is more detrimental to persons who carry the *GSTM1* null genotype. Well-designed, population-based studies of adequate size are needed to investigate the possible gene–gene or gene–environmentinteractions in the association between gene polymorphisms and CHD risk.

## Supplementary Material

Supplementary Table S1

## Data Availability

The data used to support the findings of the present study are available from the corresponding author upon request.

## Abbreviations

CHD: coronary heart disease
CI: confidence interval
GST: glutathione-S-transferase
GSTM1: glutathione S-transferase M1
NOS: Newcastle–Ottawa Scale
OR: odds ratio
ROS: reactive oxygen species
