# Supplementary material for: An updated meta-analysis showed smoking modify the association of GSTM1 null genotype on the risk of coronary heart disease
Source: Biosci Rep. 2021 Feb 12;41(2):BSR20200490. doi: 10.1042/BSR20200490 (PMC7881159; doi:10.1042/BSR20200490)
Supplement: Supplementary Table S1 [file BSR-2020-0490_supp.pdf]

Supplementary table 1. Methodological quality of studies included in the final analysis based on the Newcastle–Ottawa Scale.

Study	Selection (score)				Comparability (score)	Exposure (score)			
	Adequate definition of patient cases	Representativeness of patients cases	Selection of controls	Definition of controls	Control for important factor or additional factor	Ascertainment of exposure (blinding)	Same method of ascertainment for participants	Non-response rate	Total Score[a]
Evans[16]	1	1	1	1	0	0	1	0	5
Wilson[17]	1	1	1	1	2	0	1	1	8
Li [18]	1	1	1	1	2	0	1	1	8
Wang [20]	1	1	1	0	2	0	1	0	6
Salama[19]	1	1	1	1	2	0	1	0	7

Wilson[24]	1	1	1	1	2	0	1	0	7
Palmer[23]	1	0	1	0	2	0	1	0	5
Olshan[22]	1	1	1	1	2	0	1	1	8
Masetti[21]	1	1	1	0	2	0	1	0	6
Girisha[25]	1	1	1	1	2	1	1	0	8
Tamer[26]	1	1	1	1	2	0	1	0	7
Hayek[28]	1	0	1	1	2	0	1	1	7
Abu-Amero [27]	1	1	1	0	2	0	1	0	6
Cornelis[29]	1	1	1	0	2	0	1	1	7
Kim[30]	1	1	1	0	2	0	1	0	6
Wang[31]	1	1	1	0	2	0	1	0	6
Martin[34]	1	1	1	1	2	0	1	0	7
Manfredi	1	0	1	0	2	0	1	0	5

[33]									
Maciel[32]	1	1	1	1	2	0	1	0	7
Ramprasath [39]	1	0	1	0	2	0	1	0	5
Bazo[35]	1	0	1	0	2	0	1	0	5
Singh[40]	1	1	1	1	2	0	1	0	7
Nomani[37]	1	1	1	0	2	0	1	0	6
Norskov CCHS[38]	1	1	1	1	2	0	1	1	8
Norskov CGPS[38]	1	1	1	1	2	0	1	0	7
Norskov CIDHS[38]	1	1	1	1	2	0	1	0	7
Zhang [36]	1	1	1	1	2	0	1	0	7
Taspinar[44]	1	1	1	1	2	0	1	0	7

Kariz[41]	1	0	1	0	2	0	1	0	5
Lakshmi[42]	1	1	1	1	2	0	1	0	7
Phulukdaree [43]	1	1	1	1	2	0	1	0	7
Cora[45]	1	1	1	1	2	0	1	0	7
Yeh[46]	1	1	1	0	2	0	1	0	6
Kadıoğlu [48]	1	1	1	1	2	0	1	0	7
Bhat[47]	1	1	1	1	2	0	1	0	7
Mir[49]	1	1	1	1	2	0	1	0	7
Bhatti[50]	1	1	1	1	2	0	1	0	7

[a] Total score was calculated by adding up the points awarded in each item.
